# Exploring the care pathway for neurological conditions in a Gauteng rehabilitation hospital, South Africa

**DOI:** 10.4102/ajod.v14i0.1726

**Published:** 2025-12-12

**Authors:** Prisha Alakram-Khelawon, Sonti Pilusa, Natalie Benjamin-Damons

**Affiliations:** 1 Department of Physiotherapy, Faculty of Health Sciences, University of the Witwatersrand, Johannesburg, South Africa

**Keywords:** care pathway, neurological, rehabilitation, heads of department, patient, people with disabilities, spinal cord impairment, stroke, multidisciplinary team

## Abstract

**Background:**

People with neurological conditions resulting in disabilities face a multitude of challenges in their journey of care. Existing evidence recommends exploring region-specific care pathways to understand the multi-faceted factors that influence the health outcomes of people with neurological conditions.

**Objectives:**

To explore the care pathway for neurological conditions in a specialised rehabilitation hospital in Gauteng, South Africa.

**Method:**

This study used an explorative qualitative design. Semi-structured interviews were conducted with a purposeful sample of patients with neurological conditions and health professionals. All interviews were transcribed verbatim, and a framework analysis was used for data processing and interpretation.

**Results:**

Two overarching themes emerged: the lack of an integrated care pathway and the key role players along the care pathway. Components in the care pathway continuum included the onset and emergency management, admission into an acute facility, waiting for admission into the rehabilitation hospital, inpatient and outpatient specialised rehabilitation care and community care. Participants described role players in the care pathway.

**Conclusion:**

This study describes a detailed care pathway for people with neurological conditions in the healthcare system. Evidence suggests the lack of an integrated pathway for people with neurological conditions, especially in acute and community care settings. Multiple key components and role players are recommended for an integrated care pathway.

**Contribution:**

There is a need to develop an integrated care pathway for neurological conditions to minimise clinical risk and improve patient outcomes.

## Introduction

Worldwide, neurological conditions such as stroke and spinal cord impairment (SCI), which often result in permanent disabilities, have increased (Ding et al. 2022; Huang et al. 2023). The global report on health equity shows a 16% prevalence of people with disabilities (PWDs) and indicates an increase in global chronic diseases, which is related to neurological conditions such as stroke (World Health Organization [WHO] 2022). The South African Census 2022 reported a 6% prevalence of moderate to severe disability using the United Nations Disability Index and a 15.7% prevalence of PWD based on the broad definition of disability (Stats SA 2023a, 2024). With the increasing global burden of neurological conditions related to the years lived with disability (YLD), it is important to understand the care needs of people with neurological conditions (Feigin et al. 2020; Ferrari et al. 2024).

People with neurological conditions related to disabilities experience multifaceted health challenges that require support throughout their lives. For example, in South Africa, people with SCIs experience increased readmission rates for secondary health conditions and high mortality rates (Madasa et al. 2020; Mashola, Olorunju & Mothabeng 2019), while people with stroke experience a substantial risk of stroke recurrence and high mortality rates (Flach et al. 2020; Lekoubou et al. 2017). When considering these health challenges and inequalities experienced by PWD, a compromised quality of life seems inevitable (Gordon, Booysen & Mbonigaba 2020; Nizeyimana, Joseph & Phillips 2022a).

To improve the overall quality of life, access to rehabilitation for people with neurological conditions such as stroke and SCI remains crucial. Rehabilitation is defined as a multimodal, patient-centred care pathway to optimise function and re-integrate a person back to their home, family, society and gainful employment over a continuum (Maart et al. 2024; Negrini et al. 2022). The health system in South Africa shows significant gaps in rehabilitation (Morris et al. 2021; Visagie & Swartz 2018) care despite the Global Rehabilitation Agenda of 2030 (WHO 2017). Rehabilitation 2030 aims to strengthen and integrate rehabilitation across the health sector. In South Africa, rehabilitation care faces challenges such as limited accessibility and integration of rehabilitation services, a lack of assistive devices and rehabilitation consumables, compounded by a shortage of rehabilitation therapists (Magaqa, Ariana & Polack 2021; Tiwari, Ned & Chikte 2020). One key action of Rehabilitation 2030 is to increase rehabilitation research for reliable data that inform decision-making for quality healthcare.

There is a paucity of rehabilitation research on the care pathway for people with neurological conditions. The care pathway is defined as an individual’s integrated health journey over a continuum (Gartner et al. 2022; Wiest, Gargaro & Bayley 2023). Gartner et al. (2022) postulate that key components of an integrated care pathway are patient and caregiver-centred approaches, defined roles and responsibilities of patients, health professionals or other stakeholders, management of the health services and a region-specific approach. Integrated care pathways can improve health outcomes and health cost savings by (Wiest et al. 2023) decreasing the length of hospital stay, readmission rates, complications and the time for a PWD to reach independence (Lee et al. 2020; Portelli Tremont et al. 2022). There is a need to explore condition and region-specific care pathways for an in-depth understanding on how to manage PWD and improve their health outcomes in South Africa. Therefore, this study explored the care pathway for people with neurological conditions at a rehabilitation hospital in Gauteng province.

## Research methods and design

### Research design

An explorative qualitative design was used to describe the care pathway for neurological conditions in a rehabilitation hospital.

### Study setting

This study was conducted at the only 24-hour specialised public rehabilitation hospital providing physical rehabilitation services in Gauteng province, as per the *South African Government, National Health Act*: Regulations relating to categories of hospitals, last approved in 2012 (Legislation in Government Gazette: R185 No.35101). Gauteng is the most populated urban region in South Africa (Stats SA 2023a), and an estimated 84% of the population in South Africa access public healthcare (Stats SA 2023b). Professional health services at the rehabilitation hospital include doctors, nursing care, physiotherapy, occupational therapy, speech and language therapy, audiology, pharmacy, dietetics, social work and psychology. Patients with neurological disabilities are referred to the rehabilitation hospital from all levels of public health care, private health facilities, family or caregiver referrals and from other provinces, as there is a shortage of specialised rehabilitation hospitals in South Africa (Louw et al. 2023).

### Study participants

Purposive sampling was used to recruit study participants. The inpatient and outpatient registers showed that 98% of the patients admitted to the rehabilitation hospital had neurological conditions and the majority of these patients had SCI or stroke. Hence all eligible adult inpatients and outpatients with stroke and SCI, who could participate in an interview process and share their lived experience, were invited to participate in our study. All heads of department (HODs) at the rehabilitation hospital were included for their work experience and expert opinion. Health professionals included doctors, nurses, physiotherapists, occupational therapists, speech and language therapists, pharmacists, dietitians, social workers and psychologists. All participants were adults above 18 years.

### Procedure

Semi-structured interviews were conducted with all participants using interview guides with opened-ended questions similar to the objectives of the study. The patient interview guide differed slightly when patients’ understanding and language preferences were considered; for example, the interview guide was translated into local languages that could be easily understood by patients. However, all participants (patients and HODs) were asked to describe their experiences and views on the care pathway for neurological conditions. Demographic data collected included age, gender and other socio-economic data.

The patient interview guide also included clinical data for people with neurological conditions such as the type of neurological condition, assistive devices required, duration of the disability, type of referral facility and waiting times to access rehabilitation care.

The purpose of the study was explained to participants in a language that they could understand, and informed consent was obtained. A research assistant was available for language translation and to help the first author with other administrative tasks. Data collection was via audio-recorded face-to-face semi-structured interviews. Two pilot interviews were conducted, and the results were deliberated with the co-authors. A procedural strategy was implemented for subsequent interviews without material changes to the interview guide. The interviews took an average of 34 min each and were conducted in a private room at the hospital. Interviews were conducted until data saturation was reached; this was verified by two co-authors. A field journal was implemented, wherein all research processes and schedules were documented for research audit requirements.

### Data analysis

The recordings were transcribed verbatim by an independent transcription service. These transcriptions were verified by the first author and the research assistant who also checked the accuracy of the interviews that required English translation from a local African language. A framework analysis (Kiger & Varpio 2020) was used for systematic data handling. The MAXQDA 24 (Analytics Pro) software was used to code the transcripts and develop themes. Familiarisation and review commenced during data collection. Continuous re-reading and familiarisation of the data with draft coding were performed inductively. The first author and co-authors analysed and separately coded the three transcripts inductively. The results were discussed, and a coding framework was developed to code the remaining transcripts. Similar codes were categorised into broader categories, and these as well as the code book were verified by the two co-authors at each stage. Data source triangulation included data from inpatients, outpatients and HOD from nine different health professions and all clinical departments at the rehabilitation hospital.

### Methodological rigour

Four-dimensional methodological rigour criterion of trustworthiness, reliability, verifiability and transferability (Forero et al. 2018) was used.

The authors and research assistants were professional rehabilitation therapists with experience in disability management. The first author and research assistant were trained in qualitative research at this university. In addition, the first author orientated the research assistant to the study and data collection materials before commencing data collection. During data analysis, a code-recode procedure was implemented, wherein coding of a segment of data was performed in various separate sessions, and the results were juxtaposed between the first author and the two co-authors.

When considering reflexivity, although the first author worked at the rehabilitation hospital, she was not involved in clinical treatment and did not know the patient participants prior to the interview. However, she did know the HOD participants and therefore reassured them that she was a physiotherapist conducting a post-graduate study independent of her role at the hospital. Voluntary consent to the interview was witnessed, and each HOD also had a choice of not participating in the interview or being interviewed by the research assistant without prejudice. The first author remained neutral, listened attentively and allowed HODs to present comprehensive views. She was transparent about her role in the study throughout the process and reassured the HODs that their information would remain confidential and safe. The co-authors participated in peer review and triangulation of data at each stage.

Trustworthiness was practiced where the authors’ personal or professional views were set aside to prevent bias at each stage. A research diary and audit trail were implemented, and hard copies of raw data are available on request. Data were extracted to ensure methodological rigour, as set out in the research design and procedure.

### Ethical considerations

The University of the Witwatersrand Medical Human Research Ethics Committee granted approval for the study (M220828). The Gauteng Department of Health granted permission to conduct the study at the rehabilitation hospital, and it was registered on the National Health Research Data Base (GP_202211_037).

## Results

The results section details participant demographics, overarching themes, categories, sub-categories with supporting quotations and an illustrated summary of the care pathway.

### Demographics

A total of 41 participants, 26 patients and 15 HODs were interviewed. The HOD demographics were captured with an emphasis on years of experience and representation of HODs from all hospital health professions (Table 1).

**TABLE 1:** Demographics information for heads of department.

Demographic information	*n*	%	Mean	s.d.	Range
**Category of health professional**					
Doctor	2	13.3	-	-	-
Physiotherapist	2	13.3	-	-	-
Occupational therapist	1	6.6	-	-	-
Speech and language therapist	1	6.6	-	-	-
Social worker	1	6.6	-	-	-
Pharmacist	1	6.6	-	-	-
Clinical psychologist	1	6.6	-	-	-
Dietician	1	6.6	-	-	-
Nurse	5	33.3	-	-	-
**Gender**					
Female	14	93	-	-	-
Male	1	7	-	-	-
Age (years)	-	-	46	± 9	31−62
Total Work Experience (years)	-	-	22	± 10	8−42
Rehabilitation care work experience (years)	-	-	12	± 5	1−17

s.d., standard deviation.

The patients with neurological conditions included 20 patients with SCI and 6 patients with stroke; 11 were outpatients and 15 were inpatients (Table 2).

**TABLE 2:** Demographic information for patients

Demographic information	*n*	%	Mean	s.d.	Range
**Gender**					
Female	9	35	-	-	-
Male	17	65	-	-	-
**Age (years)**					
21-30	5	19	-	-	-
31-40	6	23	-	-	-
41-50	11	42	-	-	-
51-60	2	8	-	-	-
61-70	2	8	-	-	-
**Assistive devices**					
Wheelchairs	24	92	-	-	-
Walking aids	13	50	-	-	-
Other	4	15	-	-	-
**Neurological condition**					
Stroke	6	23	-	-	-
SCI	20	77	-	-	-
**Other conditions**					
Incontinence	20	77	-	-	-
Pain	9	35	-	-	-
HIV	7	27	-	-	-
Hypertension	7	27	-	-	-
Spasms	5	19	-	-	-
Tuberculosis	3	12	-	-	-
Pressure ulcer	2	8	-	-	-
Pulmonary condition	2	8	-	-	-
Lower limb burns	2	8	-	-	-
Diabetes mellitus	1	4	-	-	-
**Patients’ employment status**					
Employed	6	23	-	-	-
Unemployed	19	73	-	-	-
Pensioner	1	4	-	-	-
**Referring health facilities**					
Central public hospital	11	42	-	-	-
Tertiary public hospital	7	27	-	-	-
Regional public hospital	4	15	-	-	-
District public hospital	1	4	-	-	-
Private health facility	3	12	-	-	-
Average waiting time for admission at the rehabilitation hospital	-	-	17.87	± 20.25	0–90 days
Average length of stay in the rehabilitation hospital	-	-	54.08	± 40.47	3–162 days
Average duration of disability from onset until interview	-	-	3.12	± 5.63	10 days–23.22 years

HIV, human immunodeficiency virus; s.d., standard deviation; SCI, spinal cord impairment.

### Themes and categories in the care pathway

In the care pathway for neurological conditions, two overarching themes emerged with various care pathway categories and sub-categories (Table 3).

**TABLE 3:** Summary of themes and categories in the care pathway.

Themes	Categories	Sub-categories
Theme 1 Lack of an integrated care pathway	Components of the care pathway	Onset and emergency management of the neurological condition
		Care at the acute hospital
		Referral and awaiting specialised rehabilitation care
		Specialised rehabilitation care for inpatients
		Specialised rehabilitation care for outpatients
		Community care
Theme 2 People and organisations in the care pathway	Role players in the care pathway	Patient
		Family or caregiver
		Health employees: health professionals, other health staff and students
		Other patients
		Government and other stakeholders
		Community
		Health system

#### Theme 1: Lack of an integrated care pathway

From the onset, patients and HODs reported gaps in the neurological care pathway throughout the continuum. These health system challenges include overall inadequate quality of care, limited health resources, limited rehabilitation services and a lack of accessible and integrated health services throughout the care pathway:

‘… it’ is my wheelchair that needed some repairs last month. When I came here, they told me that the size is not available …’ (P4, SCI, 26-years-old)

‘… if you don’t have a CT scanner working, then you have to be transferred to another hospital, which also delays the progress … and in a stroke patient, they actually can get medication done if it’s within the first six hours but unfortunately, not all patients get to the hospital in that time …’ (P31, Jane, 44-years-old)

The participants described the care pathway for people with neurological conditions, such as stroke and SCI. This included the onset and emergency management of neurological conditions, acute care, waiting for referral and admission to the rehabilitation hospital, inpatient and outpatient rehabilitation care and community care.

**Onset and emergency management of the neurological condition:** The onset and emergency management of neurological conditions is primarily based on the patient experience. Patients described symptoms experienced prior to onset, their situational experience at full onset and delays with the ambulance services.

Five patients reported symptoms of pain and weakness before the onset of the neurological condition:

‘… In March, my right leg went weak and, I had pain in my back, and I was scared to go to the hospital so, by 13th December my second leg went weak … then I went to the hospital.’ (P2, SCI, 41-years-old)

‘I got sick when I was at Tembisa and then I went home to Mpumalanga. But they did not admit me there when I went to the hospital. They didn’t see anything wrong.’ (P9, SCI, 27-years-old)

Patients reported the full onset of their condition as a traumatic and emotional event:

‘… when the car accident happened, I just found myself down … So I just ran to take my kids. After that, a man came to help me with my kids. He said that I must lay down on the ground …’ (P16, SCI, 34-years-old)

‘I was trying to get out of the bath, that is when my left foot and my hand became numb … and then I quickly I took out the prop [*drain stopper*] in the bathtub, because … the way I was lying, I might drown.’ (P11, Stroke, 42-years-old)

Because of delays in ambulance services, 16 patients used a car to be transported to the health facility, including six patients with acute spinal cord injuries:

‘… we tried calling the ambulance but unfortunately it took time …’ (P13, SCI, 24-years-old)

‘They took me to the hospital because of the ambulance was taking a long time to come.’ (P11, Stroke, 42-years-old)

**Care at the initial or acute health facilities:** Patients reported that they first consulted with primary health care or sub-acute care prior to admission or referral to an acute care facility

Patients reported consulting for care at clinics or lower-level hospitals at onset:

‘When the doctor checked me at the clinic, my blood pressure was high. So, the following day they took me to the other hospital.’ (P1, Stroke, 50-years-old)

‘… after the stabbing, the community members … they took me to the local clinic … after a while, the ambulance came, and it took me to a hospital … then at a later time, I was also referred to another hospital.’ (P4, SCI, 26-years-old)

Patients described their experience in the emergency unit, having medical tests, admission to intensive care unit (ICU), high care or a ward and limited rehabilitation services in the acute facility:

‘… I was in ICU … I moved to the ward … I spent maybe six months in hospital, because of the bedsore and operation …’ (P7, SCI, 32-years-old)

‘… I just sit on the bed all day, doing nothing … I saw the physiotherapist and the occupational therapist, maybe once or twice a week …’ (P12, Stroke, 42-years-old)

**Referral to specialised rehabilitation care:** The patients explained that a health professional completed the rehabilitation referral forms and made relevant transport arrangements for admission. There were delays in admission to specialised rehabilitation care, and five patients reported going home while waiting.

The patient was referred to specialised rehabilitation care with noted delays:

‘… my form for admission to the rehab hospital was to be completed on Tuesday … The physiotherapist did try to help … we went to the nurses, and they said they were busy … I asked the night shift staff to help complete the form, but they said that the day shift staff must fill up the form … so there was a delay to be admitted at the rehab hospital.’ (P17, Stroke, 45-years-old)

‘… there was a queue of people who were supposed to be admitted here, so I had to wait.’ (P18, Stroke, 52-years-old)

Some patients were discharged while waiting for admission to rehabilitation care. They were unable to perform self-care and had an elevated level of dependency on their caregiver:

‘… they are finding me space here [*rehabilitation hospital*] … So, they will take me out for a week and take me home. And then when there is space, they will call …’ (P1, Stroke, 50-years-old)

‘… I have never been paralysed or anything before. So, when I got home from hospital … my mother used to do everything for me and I am 41 years, so it was quite difficult for my mother to clean me …’ (P2, SCI, 41-years-old)

**Specialised rehabilitation inpatient care:** The inpatient care pathway in the rehabilitation hospital included screening, where if the patient met all the criteria, they were admitted. If not, they were returned to the referring facility. Following admission, the patients were assessed by health professionals and resume the daily rehabilitation programme. Patients described the rehabilitation programme, although the HODs provided more detail.

Part of the rehabilitation programme included meetings with the patient and multidisciplinary team (MDT). The aim of the meeting was to set functional goals to be achieved by discharge. Caregivers were invited for education and training:

‘… in the family meeting the patients’ families are invited, and the role of each team member is explained, and goal setting are set at that time with the patient … then towards the end of their rehab we will still call another family meeting … on how far we have reached …’ (P34, Lira, 41-years-old)

‘… family meetings for the patients, I mean the family will come with the patient and the whole team will also discuss the goals and expectations …’ (P36, Busi, 33-years-old)

Activities for re-integration into the home, community and work are included in the rehabilitation programme and described in detail by the HODs:

‘… We also take our patients to other events in the community … the sports field … the zoo … we even bring them to the community centre so that they should be aware that we do have persons with disability …’ (P40, Sipho, 54-years-old)

‘… Occupational therapists have very lovely patient groups … all focused on reintegration.’ (P39, Eva, 37-years-old)

The patients were allowed weekend pass outs in preparation for hospital discharge:

‘… I got pass out … it was very different to be at home because here the hospital beds, they have railings, and you know the services are wheelchair friendly but at home I had to adjust …’ (P2, SCI, 41-years-old)

‘… I was not trained for the home setting, so … I was not coping …’ (P25, SCI, 49-years-old)

The MDT plans and coordinates patients’ discharge from the rehabilitation hospital:

‘… the neuro patients, most of the time they are given the referral letter to the local clinic … we give them discharge summary … the spinal patients, they are given a return date … they come to the hospital …’ (P27, Siphe, 57-years-old)

‘… we involve our patients in preparation for discharge … patients will be given a discharge summary …’ (P40, Sipho, 54-years-old)

**Specialised rehabilitation outpatient care:** Outpatient care was described by the patients; however, more details were provided by the HODs. The outpatient department administration includes patient registration, file retrieval and appointment management. Nursing care includes vital assessment, assisting doctors during consultation, patient education, wound care and incontinence management. The outpatient pharmacy issues medication and medical consumables such as catheters and wound dressings. Outpatient consultations with other rehabilitation health professionals have also been reported:

‘… the doctor will be assessing, asking the patient how he is coping … maybe the patient is having more spasms, then maybe the doctor will increase the treatment.’ (P28, Mary, 42-years-old)

‘… So, we [*pharmacy*] give them the catheters, the dry dispensary items as needed and also the medication ….’ (P31, Jane, 44-years-old)

‘… our outpatient services … occupational therapy or physiotherapy … they come for the wheelchairs … or maybe wheelchair cushions …’ (P34, Lira, 41-years-old)

Community care includes primary health care services, community services and support:

‘… we refer the patient to the nearest clinic …’ (P29, Cath, 55-years-old).

‘… the home that I am in currently is a non-profit organisation … they just provide the place, and you must come with your own helper … I also participated in a learnership program … somewhere they are looking for people with disability …’ (P7, SCI, 32-years-old)

‘… You can just give health education to help them prevent … we educate the community at large about pharmacy and healthy living in general.’ (P37, Osa, 40-years-old)

#### Theme 2: Role players in the care pathway

Participants explained that the role players in the care pathway included patients, family, caregivers, health employees, other hospital patients, the government, other organisations such as disability stakeholders and non-profit organisations, the health system and the community (Table 4). The patient is present throughout the care pathway and is assisted by family or caregivers. Health employees include qualified health professionals, students, porters and other hospital support staff. Participants reported that interaction with other patients positively influenced their care pathway experience. The community was also described in the patients journey at the onset of the neurological condition and after discharge. Government departments provided various public services in the care pathway. Other organisations for PWD included non-profit organisations and private stakeholders. The health system was identified as a care pathway role player, responsible for the operational management of health services.

**TABLE 4:** Role players in the care pathway.

Role players	Supporting quotations
Patient	‘… It has affected my lifestyle … I was not happy with not being able to do things myself … I had to go to the graduation with my wheelchair, but I was not feeling odd … those people there were welcoming … I was wearing heels on my wheelchair [laughing].’ (P25, SCI, 49-years-old)
	‘… we just sit around and just trick our minds what will help me to sit up … one day we were doing the training of sitting by myself and … I sit there by myself. And I was very happy …’ (P12, Stroke, 42-years-old)
Family or caregiver	‘… at first, my younger sisters were willing to take care of me, but because of this illness … They just make excuses that they do not have transport money … they are not willing to take care of me. So, I just spoke with my pastors and there is someone … so she is willing to take care of me’ (P12, Stroke, 42-years-old)
	‘… initially after the accident, it was a bit difficult … with time … family support, it made a bit of a difference …’ (P20, SCI, 49-years-old)
Health employees	‘… OT, physio, dietitian … all the non-clinical and clinical they also assist … porters and the cleaners because they play a role …’ (P40, Sipho, 54-years-old)
	‘… students who were assisting us to learn how to use the wheelchair …’ (P20, SCI, 49-years-old)
Other patients	‘Many times, the patients helped to feed me’ (P3, SCI, 61-years-old)
	‘… for example, when we have motivational speakers … sharing their experience …’ (P32, Siba, 47-years-old)
Government	‘… now you use public transport … That is when you will see the challenges’ (P20, SCI, 49-years-old)
	‘I am getting a disability grant from SASSA’ (P8, SCI, 51-years-old)
Other support organisations	‘ … we try to involve ourselves as a hospital with other NGOs, where we can bring other skills even via the occupational therapy …’ (P40, Sipho, 54-years-old)
	‘… they contacted me and arranged for me to get a space, at a certain self-help centre … That is where I started to apply for learnerships [*for skills development*]’ (P24, SCI, 33-years-old)
	‘… The home that I’m in currently is in it is called QASA [*SCI non-profit organisation*] … they just provide the place, and you have to come with your own helper’ (P7, SCI, 32-years-old)
Community	‘When I first went home … so people were coming … and then the church people came and give prayers …’ (P13, SCI, 24-years-old)
	‘… real life begins when you are outside. In your street you just notice that you are alone’ (P21, SCI, 46-years-old)
The health system	‘… consumables, they are scarce in clinics and stuff. Some wheelchairs that we issue here, so they are not able to get them anywhere else’ (P34, Lira, 41-years-old)
	‘… it took me 11 months being in the hospital … instead of me to spend maybe six months in hospital, because of the bedsore and operation … whenever I have to go home, they will postpone …’ (P7, SCI, 32-years-old)

### Results summary

**FIGURE 1:**
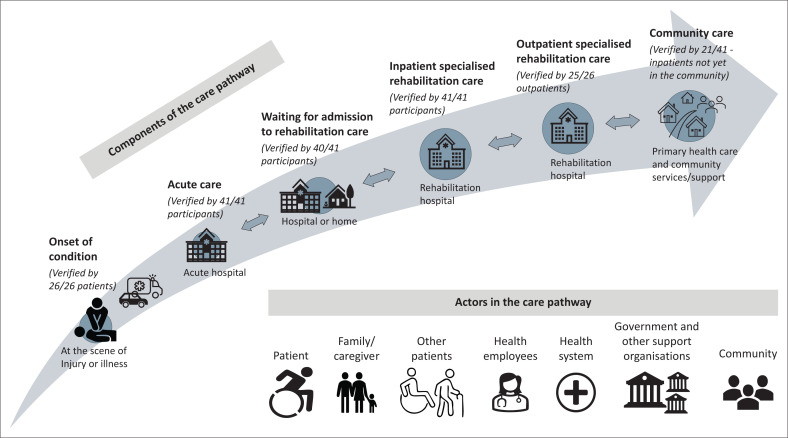
Summary of the care pathway for neurological conditions.

A summary of the results is presented in Figure 1 as an illustration of the care pathway components and role-players throughout the continuum of care required.

## Discussion

This study explored the care pathway for people with neurological conditions. The care pathway description included various components and role players from the onset, emergency management, acute care, rehabilitation care and community care. To the best of our knowledge, this is the first study to explore the care pathway continuum for neurological disabilities and provides a situational analysis on current practice in South Africa.

The findings suggest a lack of an integrated care pathway to guide the holistic and continuous care of people with neurological conditions, such as stroke and SCI. Maseko, Adams and Myezwa (2024), in a study on rehabilitation service models in primary health care, similarly found a lack of integrated rehabilitation services and gaps in the referral between levels of care. This highlights poor access to health care, limited rehabilitation services and inequities in socio-economic factors as found in prior studies (Maart et al. 2024; Morris et al. 2021). Although a stroke care pathway was outlined previously by Grimmer et al. (2018) and Perry et al. (2018), our study goes further to describe an in-depth care pathway as experienced by patients with neurological conditions over a continuum, while providing valuable input from various HODs.

The care pathway started from the onset, emergency management, acute care, rehabilitation care, as well as community care, and some of these care pathway components and role players are described in other studies. For example, the model of care for traumatic SCI, described by Wiest et al. (2023), placed emphasis on the use of the MDT, early intervention and continuous care. Gartner et al. (2022) identified patient and caregiver centric care, positioning of actors and operational management of health systems for an integrated pathway. Our study is unique as it provides a comprehensive description of specialised rehabilitation care, in the care pathway for neurological conditions in a South African health system amid the high quadruple burden of disease (Pillay-van Wyk et al. 2016).

At the start of the care pathway, patients recounted the initial and emergency management. Patients described symptoms experienced prior to full onset, where it was found that while some patients delayed health consultation because of lack of health education. Other patients did consult with health professionals, but there was delayed diagnosis or treatment, and their condition deteriorated over time. Current evidence suggests that early diagnosis and treatment can prevent or minimise the severity of the functional disability (Badhiwala et al. 2022; Galloway & Parker 2018). Community education and patient-centred care are thus recommended for early identification of spinal cord infection and stroke (Khalil & Lahoud 2020; Lurie et al. 2021).

At full onset, 16 (62%) of patients used a car to be transported to the health facility because of ambulance delays. Ambulance delays were also outlined in a previous study (Owolabi et al. 2023). Alarmingly, eight (50%) patients with traumatic spinal cord injuries were transported from the scene of injury via car and not by emergency ambulance. Stabilisation of the spine is crucial in minimising neurological fallout (Linsler & Reyes Medina 2021; Shank, Walters & Hadley 2017). When considering the onset and emergency management of neurological conditions, the priority is prevention, preservation of neurological function and minimising complications to reduce healthcare costs (Battistuzzo et al. 2016; Bharatan et al. 2021). The urgent responsiveness of the emergency ambulance service is recommended for an integrated care pathway. At onset and emergency management of neurological conditions, patients described their experience as traumatic and emotional when they reflected on how their disability had impacted their lives. This experiential recommendation for psychosocial support is also supported by other studies (Brazeau & Davis 2018; Cao et al. 2017).

Within the acute setting, patients’ experiences included the emergency department, ICU, high care and wards. Common threads that emerged were delays in surgery, sub-standard care by health professionals, nil or limited treatment received from rehabilitation health professionals and delays in referral to the rehabilitation hospital. There is little evidence of patient and family-centred care as recommended by Havana et al. (2023) or the implementation of rehabilitation protocols to optimise patient outcomes (Maciel Barbosa et al. 2023). Patients reported limitations and delays in receiving physiotherapy and occupational therapy at acute care, whereas existing studies confirm that these primary rehabilitation therapies improve functional outcomes in people with neurological conditions (Duan et al. 2021; Rahayu et al. 2020).

Referral to the specialised rehabilitation hospital was delayed, and the average waiting period for admission was 17 days. Delays were attributed to procrastination by nurses in completing referral forms and organising transport. Rehabilitation should commence early, after stabilisation of a SCI or stroke, to achieve better functional outcomes (Herzer et al. 2016; Otokita et al. 2021). Five patients were discharged while waiting for admission to the rehabilitation hospital. These patients’ experience of homestay before admission to specialised rehabilitation care suggests challenges because of a gross lack of their own functional independence and the elevated level of dependency on the caregiver. Similar homestay challenges were described by Govender et al. (2019) and Moran, Barclay and Lannin (2024), who recommended in-hospital rehabilitation care prior to discharge. The authors further recommend that the current referral pathway be reviewed with consideration of an electronic referral system for a seamless flow of patients between health facilities (Fernández-Méndez et al. 2020).

At the specialised hospital, there was evidence of more integrated (Rohwer et al. 2023) care, that is, patient- and family-centred care, and programmes to improve functionality and re-integration (Rohwer et al. 2023). The MDT consisted of health professionals, as outlined in the model of teamwork by Langhammer et al. (2017). Patient goals were monitored over time with considerations of the effectiveness of goal setting (Crawford et al. 2022; Olaleye & Agoro 2024), and family meetings aimed to educate the caregivers. Re-integration activities included home pass outs, social outings, sports programmes and vocational rehabilitation (Becker et al. 2022; Walsh et al. 2015). The care pathway at the rehabilitation hospital suggests that patients experienced various secondary and related conditions (Cao, DiPiro & Krause 2020; Richardson et al. 2021). Therefore, specialised services are recommended for incontinence care, wound care, the management of pain and spasms throughout the care pathway.

The care pathway at the rehabilitation hospital showed deliberate planning to allow adjustment to the home and community (Moran et al. 2024). However, the experiential evidence suggests major gaps in the care pathway with limited community care in primary health care, community services and support for PWD. Participants recommended more resources in community care in the form of primary health care rehabilitation services, care facilities for PWD, public education on health prevention and disability awareness in community care (Magaqa et al. 2021; Nizeyimana et al. 2022b).

Evidence identifies patients, family or caregivers, health employees, other patients, government departments, organisations that support PWD, the community and the health system as key role players in the care pathway. Gartner et al. (2022) identified similar players; however, their roles in the care pathway differed and were more region-specific. For example, Gartner et al. did not identify peer support and the role of other patients. Other patients positively influenced the care pathway as they provide physical assistance, informal motivation and formal peer support sessions, which improved their participation and disposition (Magasi & Papadimitriou 2022). In addition, at the rehabilitation hospital, patient and family-centred care was evident in goal setting, family meetings, re-integration activities and discharge planning, which facilitated patient autonomy and an individualised care pathway (Aubert, Kletz & Sardas 2023; Cameron 2021).

Health system employees and services were also key role players that influenced the care pathway. Patient interviews suggested that the MDT in the rehabilitation care pathway was a positive experience (Kinoshita, Abo & Okamoto 2020; Portelli Tremont et al. 2022). However, health professionals’ poor attitudes were reported throughout the care pathway, especially in acute care (Wong et al. 2021; Zeydi et al. 2022). Participants reported that government departments provided limited community services and support for PWD. Although social disability grants applications were aimed at assisting PWD with their increased health expenses, caregiver obligations and challenges of unemployment post-disability, the approval of these grants was a prolonged process and did not cover all expenses (Govender et al. 2015).

Other support organisations such as non-profit organisations for PWD provided limited support services such as skills training and learnership programmes, making overall integration difficult for PWD (Dorstyn et al. 2023). Accessible transport for PWD was also challenging throughout the care pathway (Pacheco Barzallo, Oña & Gemperli 2021), compounding the overall lack of community reintegration (Ahmed et al. 2018; Nizeyimana, Joseph & Phillips 2022b). Therefore, it is recommended that the government establishes a defined package of community care for PWD using multisectoral collaboration for an integrated care pathway. Maart et al. (2024) highlighted the need for key policy changes to facilitate equitable rehabilitation services and resources at all levels of care.

## Conclusion

Our study details the care pathway for patients with neurological conditions over a continuum. The multiple key role players in the care pathway were patients, family, caregivers, health employees, other patients, government departments, organisations that support PWD, the community and the health system. Evidence suggests an overall lack of an integrated care pathway for neurological conditions, especially in acute and community care. This is concerning when considering the burden of disease in South Africa (Bradshaw et al. 2019; GBD 2021 2024). For a more integrated care pathway at all levels of care, the authors recommend a defined package of care for neurological conditions using the collaboration of key components and role players identified in this study.

### Limitations

The patient participants were recruited from those accessing services at the Gauteng Rehabilitation Hospital, as this was part of a larger sequential multi-method study. Although this Gauteng Rehabilitation Hospital receives patients from all levels of care, throughout Gauteng province, a future study can be undertaken at collective health facilities or in the community. A larger range of neurological conditions, beyond stroke and SCI, should be included in future studies.

### Implication for practice

There is a need to develop an integrated care pathway for neurological conditions to minimise clinical risk and improve patient outcomes. Implications for key health service areas include preventative care and early diagnosis prior to full onset of the neurological condition, alleviating delays or limited access to emergency ambulance services. There is a need to provide access to rehabilitation care throughout the care pathway without delay, as well as specific services for PWD in primary health care and for community integration. Implementation of care packages and protocols for neurological conditions related to disabilities is required throughout the care pathway.
